# Responses of bimetallic Ag/ZnO alloy nanoparticles and urea on morphological and physiological attributes of wheat

**DOI:** 10.1049/nbt2.12048

**Published:** 2021-04-16

**Authors:** Maria Ehsan, Naveed Iqbal Raja, Zia‐ur‐Rehman Mashwani, Muhammad Ikram, Efat Zohra, Syeda Sadaf Zehra, Fozia Abasi, Mubashir Hussain, Muhammad Iqbal, Nilofar Mustafa, Asad Ali

**Affiliations:** ^1^ Department of Botany PMAS Arid Agriculture University Rawalpindi Rawalpindi Pakistan; ^2^ Department of Botany The Islamia University of Bahawalpur Bahawakpur Pakistan

## Abstract

Wheat (*Triticum aestivum* L.) is the most important staple food crop globally. According to economic survey 2018‐19, agriculture sector of Pakistan grew by 0.85%, with wheat accounting for 8.9% of agriculture and 1.6% of GDP, and its production fell short of the target by 4.9%. Wheat requires beneficial ties to improve its efficiency with the help of modern technology. Nanotechnology modifies conventional agricultural practices as these are stimulating agents for plant growth. Green bimetallic Ag/ZnO alloy nanoparticles (NPs) synthesised from salts reduced by *Moringa oleifera* and characterised by UV‐visible spectroscopy, scanning electron microscopy, and energy‐dispersive X‐ray spectroscopy are studied herein. Different concentrations of urea and Ag/ZnO alloy NPs were applied exogenously to wheat plants (Pakistan‐13 and Galaxy13). A significant effect of 100 mg/L urea and 75 ppm Ag/ZnO alloy NPs was observed on the morphology of wheat, with a maximum increase of 58% plant length, 85% leaf area, 89% plant fresh weight and 76% plant dried weight. In physiological parameters, relative water content and membrane stability index have shown maximum increases of 39% and 77%, while chlorophyll a, b, and total chlorophyll content (TCC) showed maximum increases of 92%, 71%, and 84% respectively. Evidence of the morpho‐physiological responses of urea and green synthesised alloy NPs on wheat varieties are reported on.

## 1 INTRODUCTION

*Triticum aestivum* L. belongs to the Poaceae family, and is commonly known as wheat, and is a staple food and the third most common crop in the world [1]. According to the Government of Pakistan, growth of wheat was 25.195 million tons, the sector grew by 0.85%, lower than the target of 3.8%, in 2018–2019. Humanity is facing major challenges to meet the demand for food production [2]. Fertilisers provide essential nutrients for plant growth, thus improving development and yield and the nitrogen requirement of crop plants is greater than most others. Urea (containing 46% nitrogen) is a low‐cost quick‐action fertiliser [3]. Excessive use of nitrogenous‐based fertilisers can be harmful, so for the beneficial effects of fertilisers slow‐controlled release is of more beneficial [4]. With the help of modern technology crop plants require beneficial aids to improve their efficiency.

Nanotechnology is the 21st century modern science dealing with the production of nanoparticles (NPs) (dimensions of 1–100 nm), which are stimulating agents for improving the growth and yield of plants [5]. The biological approach to synthesis of NPs is an efficient, inexpensive, easily scaled‐up, and environment‐friendly method [6]. Nanoscale materials combined into a single structure lead to the formation of alloy NPs and bimetallic Ag/ZnO alloy NPs (consisting of two types of nanocomponents) are well‐organised NPs [7]. A simple method of preparing Ag/ZnO alloy NPs is the co‐reduction of silver and zinc precursors. The influence of synthesis conditions, nucleation, growth, and properties of bimetallic alloy NPs are more complex as compared to monometallic NPs, as two metal precursors are involved [8]. Bimetallic alloy NPs have increased surface area, resulting in enlarged functionality and novel behaviour. The electrical conductivity of silver is the highest among the metal filters and their oxides have better conductivity than many other metals [9]. Monometallic NPs have a beneficial role in crop plants as they enhance the physiological attributes, resulting in increased growth and yield of crops like wheat [10].

In this research, bimetallic Ag/ZnO alloy NPs were synthesised biologically using leaf extract and characterisation of alloy NPs was carried out. In further research, the authors exogenously applied different concentrations of alloy NPs and fertiliser to wheat and assessed the morphological and physiological attributes of wheat varieties.

## 2 MATERIALS AND METHODS

### 2.1 Biosynthesis of monometallic ZnO and bimetallic Ag/ZnO alloy nanoparticles

**FIGURE 1:**
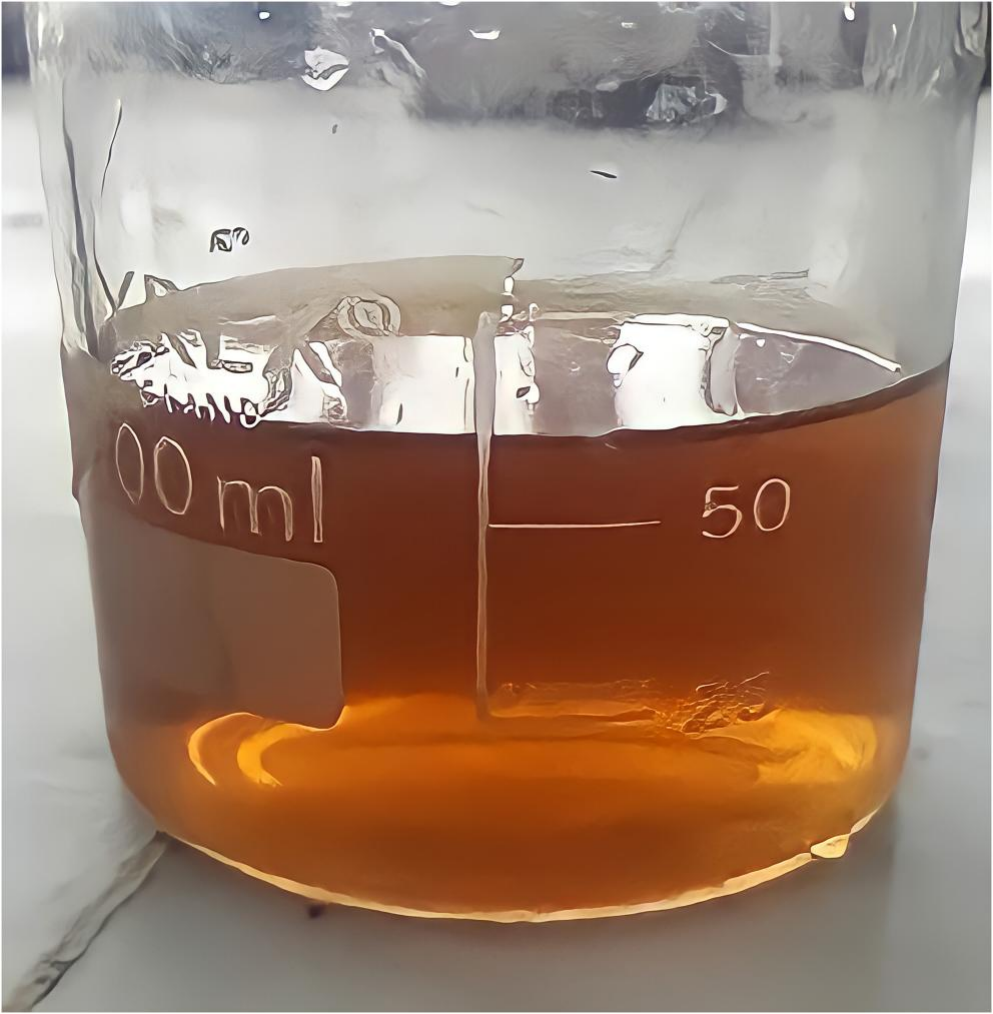
Aqueous leaf extract of *Moringa oleifera*

ZnO and Ag/ZnO alloy NPs were synthesised by the co‐reduction method described by Sorbium et al. [11]. ZnO NPs synthesis was done by reducing zinc sulphate heptahydrate by using leaf extract of *Moringa oleifera* L. Leaves were washed with water, dried, and cut into pieces. About 30 g of leaves were taken in a beaker containing 300 ml of water and allowed to boil in an oven for 15 min. The extract was filtrated thrice to remove any contaminants. Figure 1 shows the formation of aqueous leaf extract of *M. oleifera*. In 30 ml of leaf extract, 1 g of salt was added and the mixture stirred for 4 h at 60°C. The solution was then centrifuged at 4000 rpm for 20 min and the pellet was re‐dispersed with water and then with ethanol. After that, samples were dried in an oven for 6 h at 80°C and a brown powder was obtained and calcined for 4 h at 500°C to obtain a white powder. The powder was stored in a container for analysis of UV‐spectrum as a control for bimetallic NPs.

**FIGURE 2:**
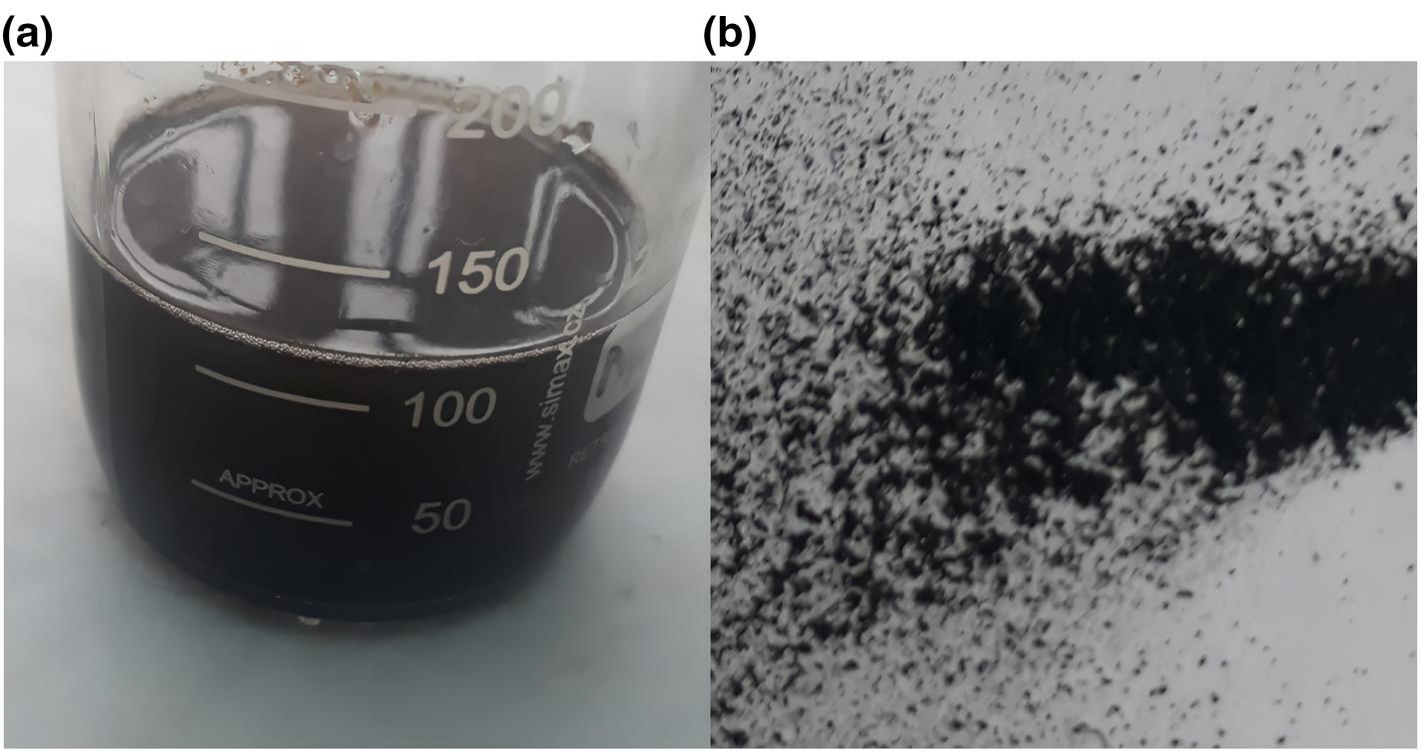
Ag/ZnO alloy nanoparticles: (a) solution form; (b) powder form

Alloy NPs of Ag/ZnO were prepared by reduction of silver nitrate and zinc sulphate hexahydrate salt by using leaf extract of *M. oleifera*. The 25 ml leaf extract was mixed with 50 ml AgNO_3_ (0.01 M) and 50 ml ZnSO_4_.7H_2_O (0.1 M) in a flask and allowed to heat for 4 h at 80°C, the colour then changed to brown (Figure 2a). After that, the solution was allowed to cool overnight at 25°C. The following day, centrifugation of the mixture was done at 4000 rpm for 25 min. The supernatant phase was discarded and the pellet phase was collected. The process of centrifugation was repeated three times with distilled water to remove any unwanted substances. A powder form was obtained by drying the NPs in a hot air oven for 6 h at 80°C (Figure 2b). The resultant Ag/ZnO alloy NPs were used for characterisation, as well as to study their responses to the morphology and physiology of wheat varieties.

### 2.2 Characterisation of biosynthesised bimetallic Ag/ZnO alloy nanoparticles

#### 2.2.1 UV‐visible spectroscopy

This tool is more appropriate for confirming surface plasmon resonance. Minute quantities of NPs were added to distilled water and suspended by sonication. UV‐visible spectra were recorded (300–700 nm) using a HALO DB‐20 spectrophotometer from Fatima Jinnah Women's University, Rawalpindi.

#### 2.2.2 Scanning electron microscopy

The morphology of bimetallic Ag/ZnO alloy NPs was observed by the SIGMA model of SEM from IST, Islamabad. A minute amount of NPs was placed on a copper grid (carbon‐coated) and blotting paper was added. For drying, films were then placed under a mercury lamp for 5 min and at different magnifications micrographs were taken by SEM.

#### 2.2.3 Energy‐dispersive X‐ray spectroscopy

For elemental analysis, NPs were dropped on a carbon film followed by drying of films, and analysis was performed using an EDX detector.

### 2.3 Exogenous application of nanoparticles and fertiliser

Sterilised seeds of two wheat varieties (Galaxy‐13 and Pak‐13) were obtained from NARC‐Islamabad and nitrogenous fertiliser (urea) from the local market of Rawalpindi. Different concentrations of fertilisers and alloy NPs were prepared and applied thrice with a gap of 2 days to wheat varieties at the trifoliate stage (foliar application). Table 1 illustrates the different treatments of alloy NPs and fertiliser on the varieties of wheat.

**TABLE 1:** The layout of the experiment with details of the different concentrations of NPs and urea

	Treatments
T_0_	Control
T1	Urea (50 mg/L) + NPs (25 ppm)
T2	Urea (50 mg/L) + NPs (50 ppm)
T3	Urea (50 mg/L) + NPs (75 ppm)
T4	Urea (50 mg/L) + NPs (100 ppm)
T5	Urea (100 mg/L) + NPs (25 ppm)
T6	Urea (100 mg/L) + NPs (50 ppm)
T7	Urea (100 mg/L) + NPs (75 ppm)
T8	Urea (100 mg/L) + NPs (100 ppm)

Abbreviation: NPs, nanoparticles.

### 2.4 Assessment of morphological attributes

For the analysis of morphological parameters, plants from each treatment were collected and the length of the plant measured using a scale. The leaf area was noted using a leaf area meter (CID, CI‐202). The fresh weight (PFW) and then the dry weight of plants (PDW) were calculated by placing them in an oven for 24 h at 65^o^C.

### 2.5 Assessment of physiological attributes

The relative water content (RWC) was measured using the method of Wheatherley [12]. The leaf fresh weight was measured before being placed in water‐filled test tubes for 1 day measurement of the for turgid weight. For dry weight measurement, samples were then placed in an oven for 7 days at 70^o^C.

$$\text{RWC}=\frac{\left(\text{Fresh}\;\text{weight}-\text{Dry}\;\text{weight}\right)}{\left(\text{Turgid}\;\text{weight}-\text{Dry}\;\text{weight}\right)}\times 100$$

The membrane stability index (MSI) was recorded using the method of Sairam [13]. Small pieces of washed leaves of each treatment were added to water‐filled test tubes, placed in a water bath at 40^o^C for half an hour, and then the electric conductivity (*C*_1_) was noted. Again test tubes were placed in a water bath at 100^o^C for 10 min for measurement of the electric conductivity (*C*_2_).

$$\text{MSI}=\left(1–C_{1}/C_{2}\right)\times 100$$

A UV‐visible spectrophotometer was used for the measurement of leaf chlorophyll content by following the method of Bruinsma [14]. For each treatment, 0.2 g leaf was ground in acetone (10 ml), filtered, and the extract placed in test tubes. At wavelengths of 645, 652, and 663 nm, the absorbance was noted.

$$\text{Chlorophyll}\;a=\left(12.7\times A_{663}\right)–\left(2.7\times A_{645}\right)$$

$$\text{Chlorophyll}\;b=\left(22.9\times A_{645}\right)–\left(4.7\times A_{663}\right)$$

$$\text{Total}\;\text{chlorophyll}\;\text{content}=\left(A_{652}\times 1000/34.5\right)$$

### 2.6 Statistical analysis

Three replicates were prepared for each treatment and the calculation of average value carried out, with the results interpreted as a standard error of mean value. Two‐way analysis of ANOVA (SPSS 16) was used for statistical analysis of experimental data at *p* < 0.05.

## 3 RESULTS AND DISCUSSION

### 3.1 Synthesis and characterisation of Ag/ZnO alloy nanoparticles

**FIGURE 3:**
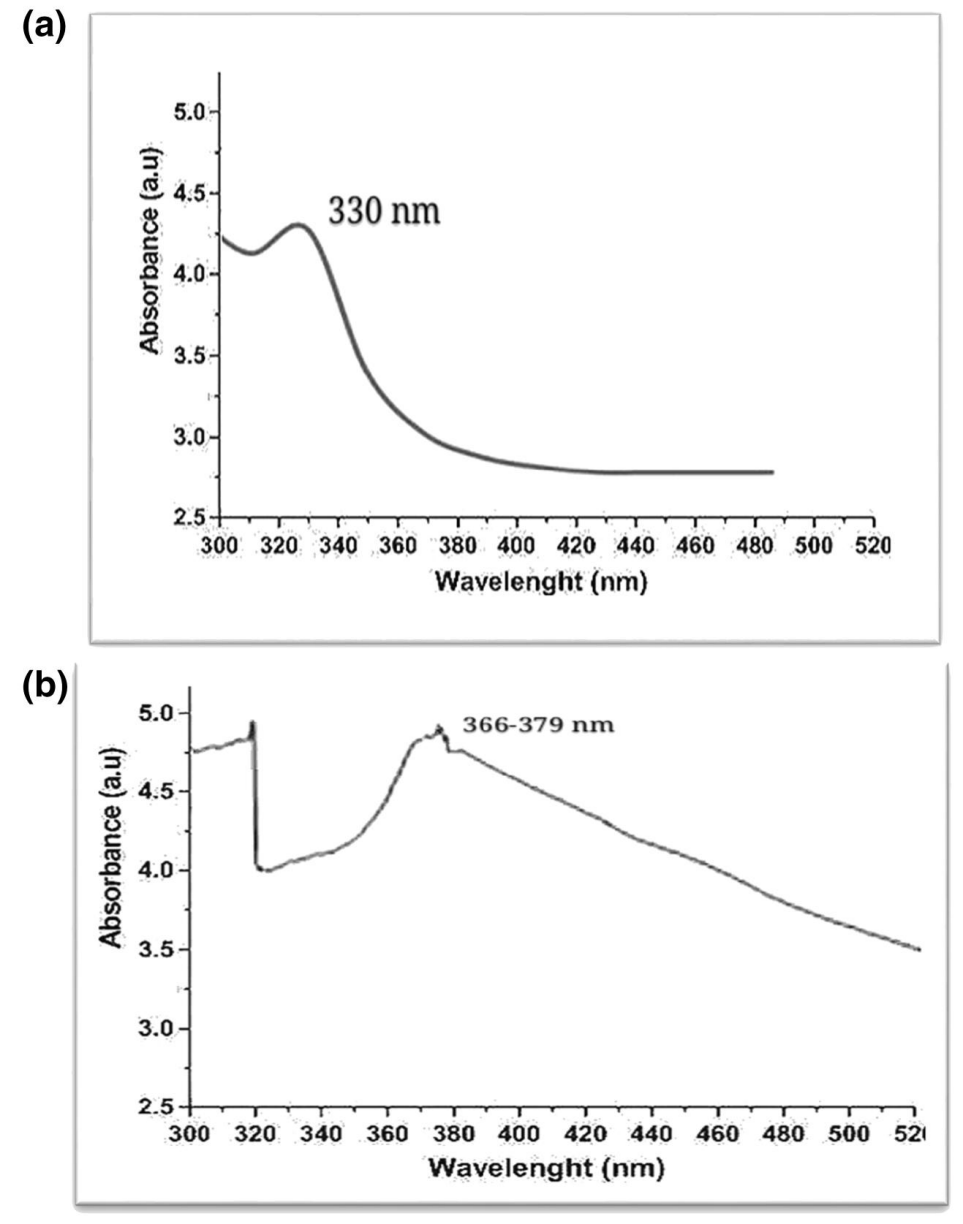
UV‐visible spectrum: (a) ZnO NPs; (b) Ag/ZnO alloy NPs. NPs, nanparticles

**FIGURE 4:**
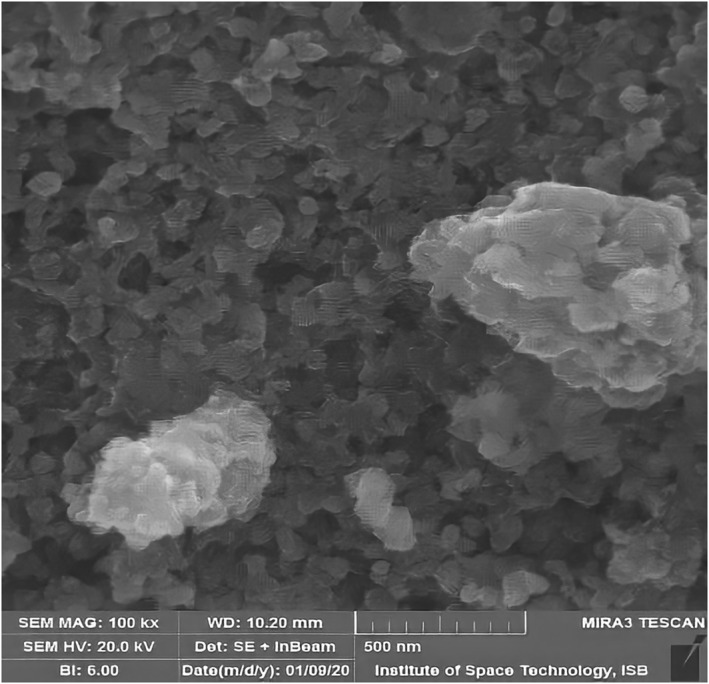
Scanning electron microscopy micrograph of the green synthesised Ag/ZnO nanoparticles

**FIGURE 5:**
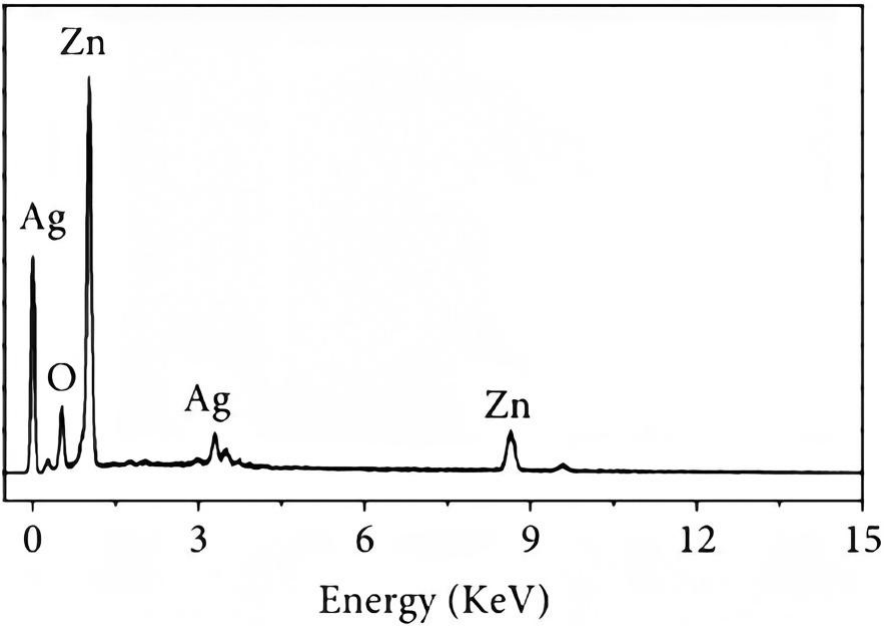
Energy‐dispersive X‐ray spectroscopy spectrum of green synthesised Ag/ZnO nanoparticles

Alloy NPs were prepared using leaf extract of *M. oleifera*. Previous studies have shown that leaf extract has certain active compounds, reduced silver nitrate, and zinc sulphate heptahydrate in Ag/ZnO NPs [15]. Alloy NPs were characterised by UV‐visible spectroscopy, SEM, and EDX. In a UV‐visible spectrum of wavelength 300–520 nm, the characterisation peak for ZnO was recorded at 330 nm (Figure 3a) and for Ag/ZnO alloy NPs it was recorded in the range of 330 nm and 366–379 nm (Figure 3b). Different characterisation peaks ranging from 360 to 410 nm confirm the synthesis of Ag/ZnO alloy NPs [16]. Using SEM, structural analysis of NPs was done. Figure 4 was taken using SEM and shows the spherical morphology of alloy NPs, with a size range between 46–66 nm. However, the SEM image could not distinguish between Ag and ZnO NPs. Particles with sizes of 1–100 nm diameter are considered to be NPs. Sorbium et al. [11] also documented similar shapes of alloy NPs. Using an EDX detector, elemental determination was done. Different peaks were monitored for zinc, silver, and oxygen ions. Figure 5 shows the highest peaks between 0.1 and 1.5 KeV using an EDX spectrum. Nagaraju et al. [17] also documented similar findings for elemental determination.

### 3.2 Assessment of morphological parameters

#### 3.2.1 Plant length and leaf area

**FIGURE 6:**
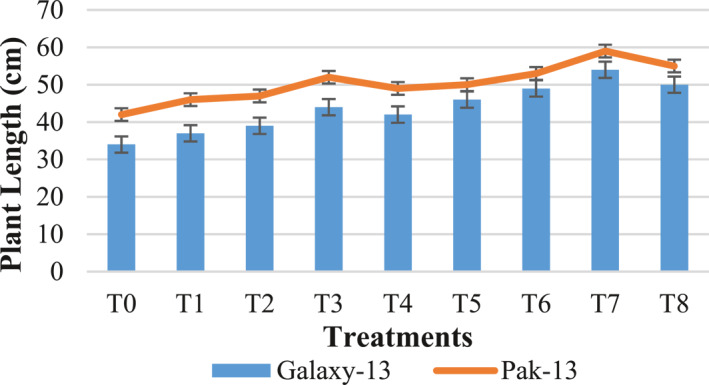
Length of wheat plants in response to different treatments

**FIGURE 7:**
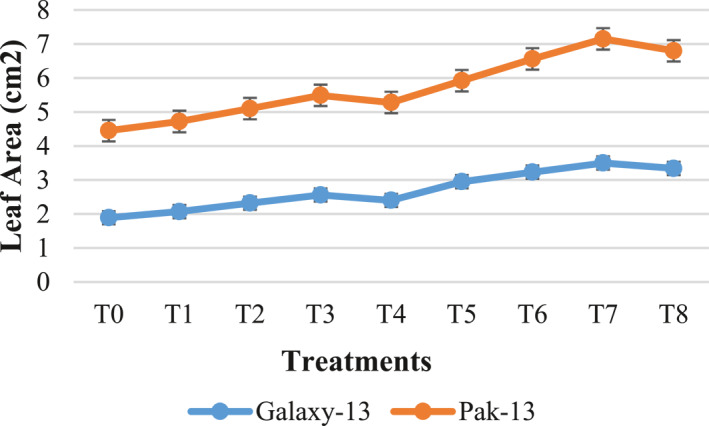
Leaf area in response to different treatments

It is illustrated in Figure 6 that the wheat variety Galaxy‐13 treated with 100 mg/L urea and 75 ppm alloy NPs shows increased plant length of about 58% and 22% as compared to controls and T3, respectively, while Pak‐13 shows 40% increased length as compared to controls and 13% as compare to T3 when treated with 100 mg/L urea and 75 ppm alloy NPs. Treatment with silver and zinc oxide NPs results in enhancement of growth parameters such as shoot and root length. In the literature, the reason behind increased length is described as increased plant expansion [18]. The best results for the leaf area were observed at 100 mg/L urea and 75 ppm NPs (Figure 7). Compared with controls, Galaxy13 and Pak‐13 show increased leaf areas of 85% and 42%, while as compared to T3 (50 mg/L urea and 75 ppm NPs) these varieties show 37% and 24% increases in leaf area, respectively. The current findings are similar to those of Yao et al. [19].

#### 3.2.2 Plant fresh and dry weight

**FIGURE 8:**
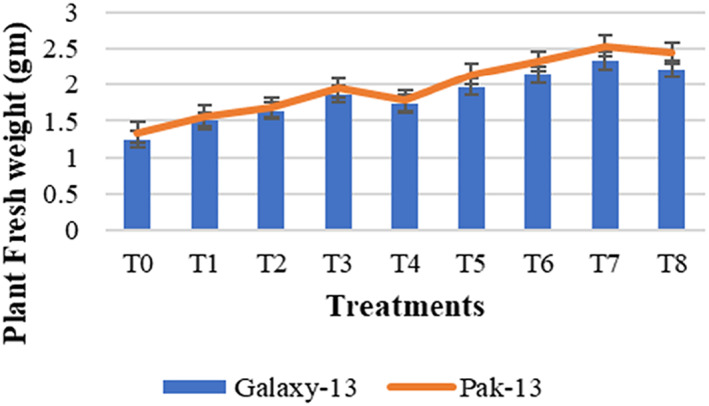
Plant fresh weight in response to different treatments

**FIGURE 9:**
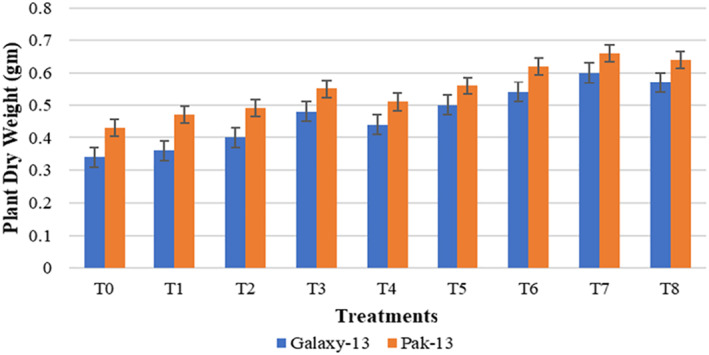
Plant dry weight in response to different treatments

Data obtained for the fresh weight of wheat plants in Figure 8 illustrate that both wheat varieties, Galaxy‐13 and Pak‐13, show increased fresh weight of 87% and 89% in T7 (100 mg/L urea and 75 ppm NPs) as compared to controls. In comparison with T3 (50 mg/L urea and 75 ppm NPs) fresh weight of Galaxy‐13 was increased by 25% and that of Pak‐13 by 30%. A remarkable increase in the dry weight of wheat was observed in T7 (Figure 9). Galaxy‐13 was increased by 76% and Pak‐13 by 53% dry weight as compared to controls. Galaxy‐13 and Pak‐13 varieties showed increased dry weights of about 25% and 20%, respectively, as compared to T3. The findings of Rafique et al. [20] are similar to this research with the application of 75 ppm concentration of NPs increasing plant biomass.

### 3.3 Physiological parameters

#### 3.3.1 Relative water content and membrane stability index

**FIGURE 10:**
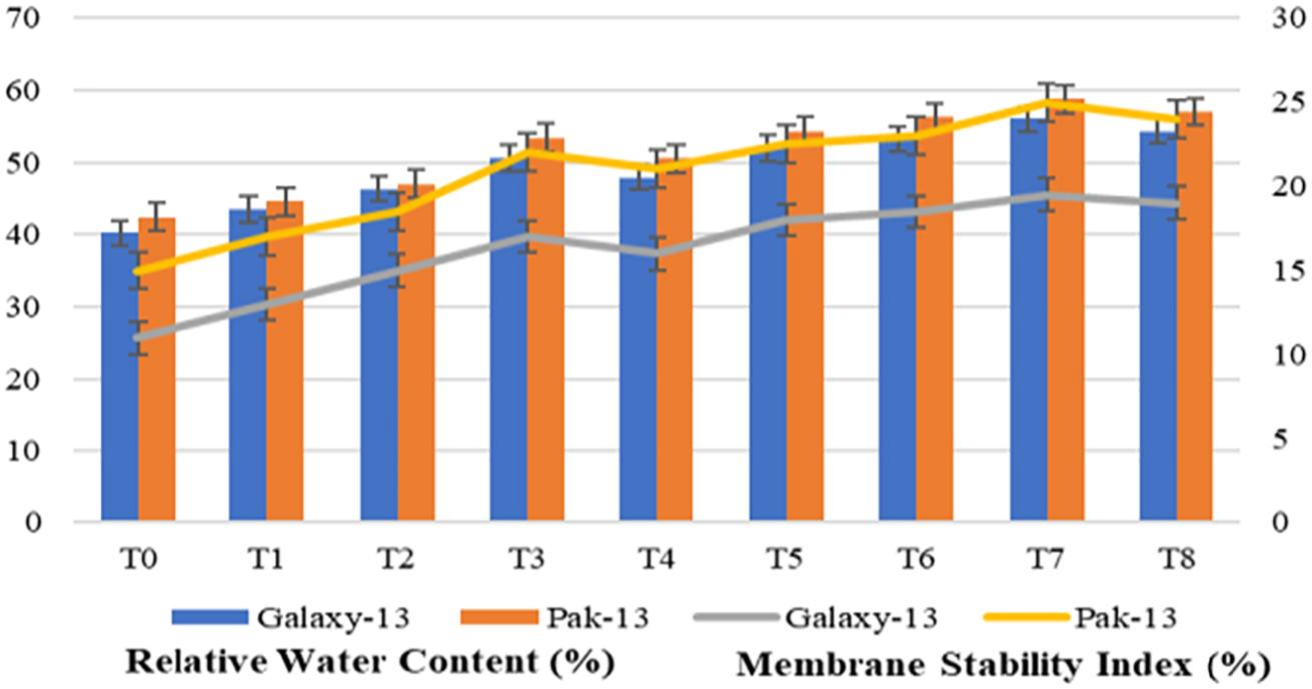
Relative water content and membrane stability index in response to different treatments

Data regarding RWC and MSI in Figure 10 illustrate that both wheat varieties showed an increase in RWC and MSI at T7 (100 mg/L urea and 75 ppm alloy NPs). Both varieties showed increased RWC of about 39% as compared to controls and 11% as compared to T3. The findings of Singh et al. [21] also confirm these results, with the RWC of plants increasing with an increase in the concentration of silver and zinc oxide NPs. Comparing with controls, Galaxy‐13 shows a 77% and Pak‐13 a 66% increase in MSI, while in comparison with T3 both varieties showed 14% increased indexes. Martinez‐Ballesta et al. [22] explained the reason for this as being that there was more solute leakage as the application of silver NPs increases MSI.

#### 3.3.2 Chlorophyll a and b content

**FIGURE 11:**
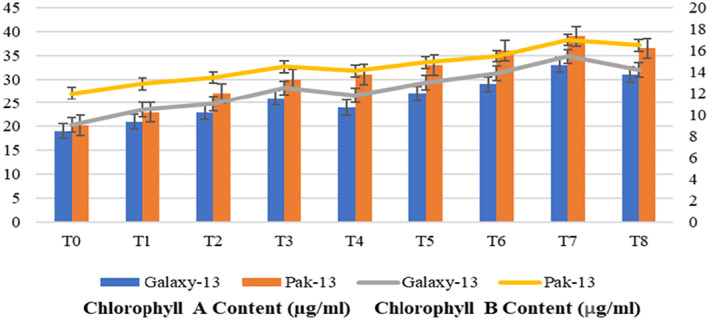
Chlorophyll a and b contents in response to different treatments

Treatment with 100 mg/L urea and 75 ppm alloy NPs shows increased chlorophyll a and b contents in wheat plants (Figure 11). There was a 73% increased chlorophyll a content in Galaxy‐13 and 92% in Pak‐13, as compared with plants treated only with water. The chlorophyll a content of Galaxy‐13 and Pak13 increased about 27% and 30%, respectively, as compared with plants treated with 50 mg/L urea and 75 ppm alloy NPs. A remarkable increase in chlorophyll b content was demonstrated at T7. Galaxy‐13 and Pak‐13 show 71% and 42% increased content as compared to controls, respectively. While comparing with T3, Galaxy‐13 and Pak‐13 exhibited 24% and 17% increased content, respectively. The authors’ results are in agreement with those of Mathur et al. [23]. Ashraf and Harris [24] explained the reason behind chlorophyll a and b content increases as being because of an increase in leaf area expansion, premature, reduced leaf senescence, reduced injury in thylakoid lamellae, and stroma paired photosynthetic machinery, hence resulting in an advancement of chlorophyll a and b biosyntheses.

#### 3.3.3 Total chlorophyll content

**FIGURE 12:**
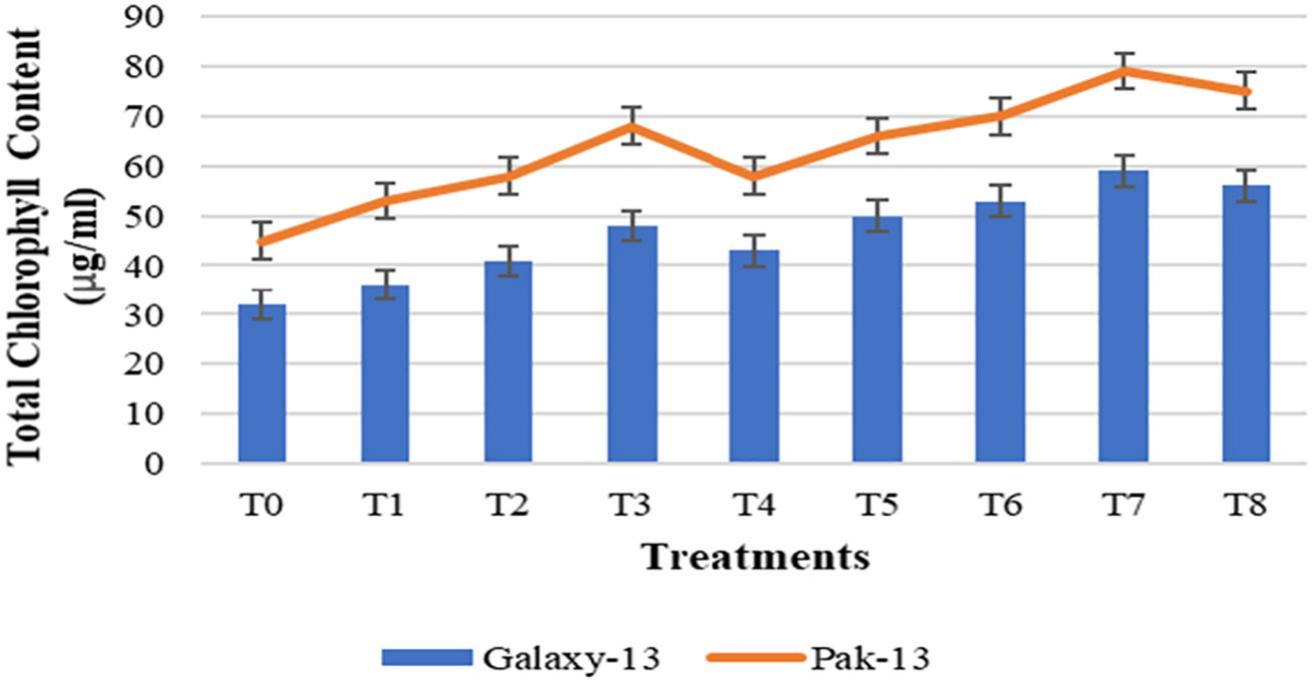
Total chlorophyll content in response to different treatments

The results of total chlorophyll content (TCC) measurement are presented in Figure 12, where the highest content (Galaxy‐13, 84%; Pak‐13, 76%) was observed with 100 mg/L urea and 75 ppm alloy NPs as compared to controls, and increased TCC was observed at about 23% in Galaxy‐13 and 16% in Pak‐13 as compared to T3. These findings are complementary to those of Sharma et al. [18] and Sun et al. [25], who concluded that silver and zinc oxide NPs enhance the TCC, which suggests that NPs might increase the numbers of proteins related to photosynthesis.

## 4 CONCLUSION

From the presented research work, it is concluded that alloy NPs synthesised from leaf extracts are eco‐friendly and less toxic. The application of green synthesised Ag/ZnO alloy NPs along with urea 100 mg/L has a significantly positive effect on wheat varieties, with improved growth (morphological and physiological attributes). Morphological characteristics (plant length, leaf area, PFW and PDW) showed their optimum increase at 100 mg/L urea and 75 ppm Ag/ZnO NPs. The maximum effects for RWC, MSI, chlorophyll a, b, and TCC were also found at 100 mg/L urea and 75 ppm NPs. Once NPs entered plant cells, they had long‐lasting effects on plant growth. Very few studies have been conducted regarding the application of bimetallic alloy NPs. However, there is a need to study the biochemical and yield attributes of wheat varieties in response to urea and Ag/ZnO alloy NPs for their application at a commercial level. Furthermore, the molecular study of plants also needs to be explored further.
